# Biogeneration of silver nanoparticles from *Cuphea procumbens* for biomedical and environmental applications

**DOI:** 10.1038/s41598-022-26818-3

**Published:** 2023-01-16

**Authors:** María G. González-Pedroza, Andrea Regina Tapia Benítez, Saúl A. Navarro-Marchal, Eduardo Martínez-Martínez, Juan A. Marchal, Houria Boulaiz, Raúl A. Morales-Luckie

**Affiliations:** 1 Departamento de Biotecnología, Facultad de Ciencias, Universidad Autónoma del Estado de México (UAEMex), 50200 Toluca, Mexico; 2 Facultad de Química, Universidad Autónoma del Estado de México (UAEMex), 50200 Toluca, Mexico; 3 Instituto de Biopatología y Medicina Regenerativa (IBIMER), Universidad de Granada, 18016 Granada, Spain; 4 Instituto Nacional de Medicina Genómica (INMEGEN), Arenal Tepepan, Tlalpan, 14610 Ciudad de México, Mexico; 5 Departamento de Física Aplicada, Facultad de Ciencias, Universidad de Granada, 18071 Granada, Spain; 6 Instituto de Investigación Biosanitaria Ibs. GRANADA, Hospitales Universitarios de Granada-Universidad de Granada, 18012 Granada, Spain; 7 Research Unit “Modeling Nature” (MNat), University of Granada, 18016 Granada, Spain; 8 Departamento de Anatomía Humana Y Embriología, Universidad de Granada, 18016 Granada, Spain; 9 Centro Conjunto de Investigación en Química Sustentable UAEMex-UNAM (CCIQS), Universidad Autónoma del Estado de México (UAEMex), 50200 Toluca, Mexico

**Keywords:** Cancer, Nanoscience and technology

## Abstract

Nanotechnology is one of the most important and relevant disciplines today due to the specific electrical, optical, magnetic, chemical, mechanical and biomedical properties of nanoparticles. In the present study we demonstrate the efficacy of *Cuphea procumbens* to biogenerate silver nanoparticles (AgNPs) with antibacterial and antitumor activity. These nanoparticles were synthesized using the aqueous extract of *C. procumbens* as reducing agent and silver nitrate as oxidizing agent. The Transmission Electron Microscopy demonstrated that the biogenic AgNPs were predominantly quasi-spherical with an average particle size of 23.45 nm. The surface plasmonic resonance was analyzed by ultraviolet visible spectroscopy (UV–Vis) observing a maximum absorption band at 441 nm and Infrared Spectroscopy (FT IR) was used in order to structurally identify the functional groups of some compounds involved in the formation of nanoparticles. The AgNPs demonstrated to have antibacterial activity against the pathogenic bacteria *Escherichia coli* and *Staphylococcus aureus*, identifying the maximum zone of inhibition at the concentration of 0.225 and 0.158 µg/mL respectively. Moreover, compared to the extract, AgNPs exhibited better antitumor activity and higher therapeutic index (TI) against several tumor cell lines such as human breast carcinoma MCF-7 (IC_50_ of 2.56 µg/mL, TI of 27.65 µg/mL), MDA-MB-468 (IC_50_ of 2.25 µg/mL, TI of 31.53 µg/mL), human colon carcinoma HCT-116 (IC_50_ of 1.38 µg/mL, TI of 51.07 µg/mL) and melanoma A-375 (IC_50_ of 6.51 µg/mL, TI of 10.89 µg/mL). This fact is of great since it will reduce the side effects derived from the treatment. In addition, AgNPs revealed to have a photocatalytic activity of the dyes congo red (10^–3^ M) in 5 min and malachite green (10^–3^ M) in 7 min. Additionally, the degradation percentages were obtained, which were 86.61% for congo red and 82.11% for malachite green. Overall, our results demonstrated for the first time that *C. procumbens* biogenerated nanoparticles are excellent candidates for several biomedical and environmental applications.

## Introduction

Nanotechnology is currently widely used in different areas^1^ such as solar energy conversion^2^, catalysis^3^, medicine^4^, and water treatment^5^. Some specific applications of nanotechnology in biotechnology^6^ and biomedicine^7^ include additives for textile industry^8^, food packaging^9, 10^, protein immobilization^11^, and development of optoelectronic materials^12^. Due to its versatility, there is an increasing interest in designing new methods for nanoparticle synthesis and modification or functionalization of its surface to improve their effectiveness. Green synthesis has shown a great potential because it is inexpensive and easy to reproduce method. Moreover, it has several applications because chemical elements in their nanometric or nanoparticle (NP) form have different properties than those properties manifested on the micro or macroscopic scale or even in their ionic form^13–15^. One of the most widely used nanoparticles, both at the industry and biomedical level, are silver and gold nanoparticles. Specifically silver nanoparticles play an important role in the field of biology and medicine^16, 17^ due to their attractive antimicrobial, antibacterial, antifungal^18, 19^, anticoagulant^20^, thrombolytic^20^, antidiabetic^21^, antioxidant^22^ and antiviral properties^13^.

Silver nanoparticles can be generated through several methods^23^, but the most common include physical, chemical and biological components. Although it is true that the green synthesis has three main components: a metal precursor^24^, a reducing agent^25^ and a stabilizing agent^26^, it could be said that it is a derivative of both methods (chemical and biological). One of the fundamental pillars of biosynthesis is to obtain extracts with high antioxidant power such as polyphenols^24^, reduced sugars, nitrogenous bases and amino acids; capable of reducing cations in a metal salt solution. Silver nanoparticles show great reactivity with enzymes, DNA and RNA^27^, caused by the interactions with thiol, carboxylate, phosphate, hydroxyl, imidazole, indole or amine groups, triggering a series of reactions that prevent the formation of microbial processes^1^. It is important to recognize that there is a wide variety of biological resources that can be used for green or ecological synthesis, such as microorganisms (bacteria^28, 29^, fungi^29^, algae^30^), plants and their derivatives^31^, animal metabolites and even organic waste^32^, which opens up a range of possibilities to generate nanoparticles as well as to apply them in various areas.

Recently, AgNPs have been widely used to degrade organic dyes through redox potential techniques and photocatalytic reaction under solar radiation^33^. In addition, AgNPs are used as an antimicrobial agents for a wide range of microorganisms and their use as cytotoxic agent against cancer cells has also become popular in recent years^34^. Some plant extracts have a potent antibacterial activity against a variety of bacteria, including *E. coli* and *S. aureus*^6^. These findings highlight the importance of synthesizing nanoparticles from plant extracts. The proposed mechanism of action of AgNPs includes the attraction between positive charge of Ag ions and negative charge of the bacterial membrane which results in the alteration of the cell membrane. Moreover, Ag ions affect the activity of vital enzymes including those related to DNA replication. The anticancer activity of AgNPs has been related to the induction of cell apoptosis through the generation of reactive oxygen species (ROS)^34^.

The genus *Cuphea* comprises more of 260 species that are native to the Americas distributed from Mexico to Brazil. *Cuphea procumbens* specie has oil^35^ rich in medium chain fatty acids and are used in traditional (folk) medicine for their antioxidant, antihypertensive, cytotoxic, antiprotozoal and hypocholesterolemia activities^36^. It is a peculiar herbaceous plant native to southern Mexico, which grows in a rudimentary way in cultivated fields with moist soil, roadsides, low fields prone to flooding, on the banks of rivers and in other disturbed swampy sites, it is important to note that *C. procumbens* is not an endangered species. On the other hand, *C. procumbens* is well known as a cancer herb^37^. As its name suggests, this plant has been used in the treatment of various cancers for its pain reliver properties and anti-inflammatory activities^38^. This plant has anti-inflammatory properties and it is often used to reduce the swelling caused by injuries^39^. Analgesic properties of *C. procumbens* can be used to relieve headaches or other types of pain. The phytochemical analysis of this plant has shown the presence of alacaloids, flavonoids and glycosides in extract fractions. Several studies indicate that some flavonoids have pro-oxidant actions at high doses but at low doses they have anti-inflammatory, antiviral or antiallergic effects^40^. Thus, we decided to synthesize AgNPs using *C. procumbens* extracts to potentiate the beneficial effects of this plant. We designed a protocol to obtain AgNPs that could be used in three main applications including degradation of dyes, antibacterial tests and antitumor agents.

## Results

**Figure 1:**
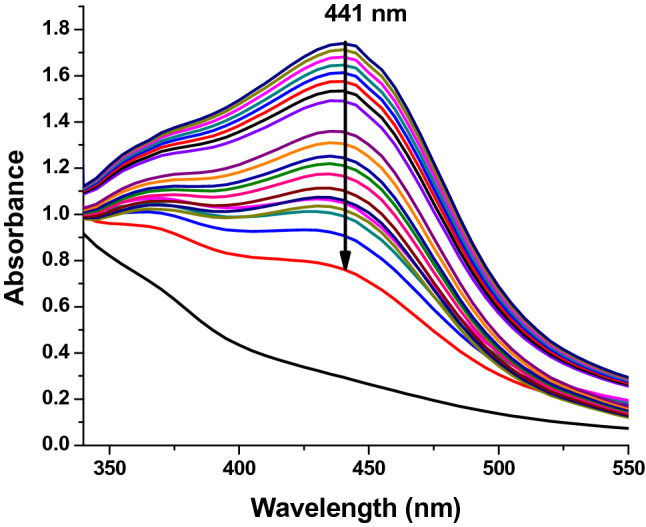
UV–vis absorption kinetic spectra of *C. procumbens* synthesized silver nanoparticles every 20 min for 6 h.

In the process of nanoparticle synthesis one of the characteristic effects is the color change from light brown to dark brown, which suggests the formation of AgNPs through extracellular activity^41^. Figure 1 shows UV spectral analysis at different time intervals. The UV–vis spectrum of the silver nanoparticles shows the monitoring of the formation of nanoparticles in a period of 6 h, recording the activity every 20 min. The UV–Vis spectrum indicated the presence of a strong and broad band in a range from 350 to 450 nm, and specifically showing a maximum absorption peak at 441 nm, which is attributed to the Surface Plasmon Resonance (SPR)^42^. This is due to the nucleation and growth of nanoparticles produced as a result of the reduction of silver ions present in the solution^43^.

**Figure 2:**
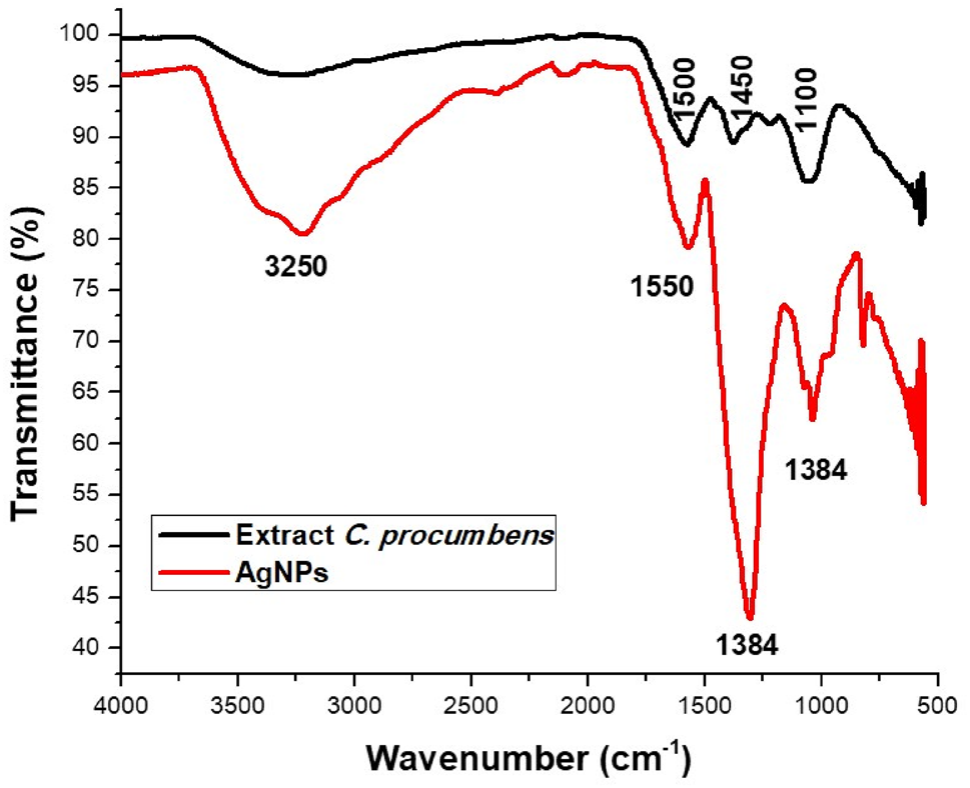
FTIR spectra of *C. procumbens* extract and AgNPs.

The aqueous extract of *C. procumbens* has bioorganic compounds bound to the surface of the AgNPs which were identified by FT-IR spectroscopy (Fig. 2) The strong absorption peaks of the leaf extract of *C. procumbens* were observed at 1500, 1450 and 1100 cm^−1^. The change in vibration around 1450 cm^−1^ of *C. procumbens* suggested the presence of aliphatic –CH and aromatic-OH groups such as hydroxy flavones and hydroxy xanthones^39^. The vibration around 1100 cm^−1^ was attributed to the primary amine C-N stretching vibrations of aliphatic amines, the presence of N–H stretching of amine groups, aliphatic C–H stretchings^44^, C–N stretching of amines/proteins and C–H stretchings, respectively and according to other authors.

The FTIR spectra of AgNPs also showed peaks at 3250, 1550, 1384, 1100 cm^−1^ due to O–H stretching of polyphenols , C=C or C=O stretch of carboxylic acids^14^, amide stretching, C–O stretching, C–N stretching of amine proteins^27^, alkene C=C stretchings, =C–H bending and bending vibration of C–H stretching^18^, respectively.

The peaks at 1550 and 1450 cm^−1^ are characteristic of primary amide stretches^45^. These functional groups are representative of the amide or polyphenol groups of *C. procumbens*, which are responsible for the formation and stabilization of AgNPs.

**Figure 3:**
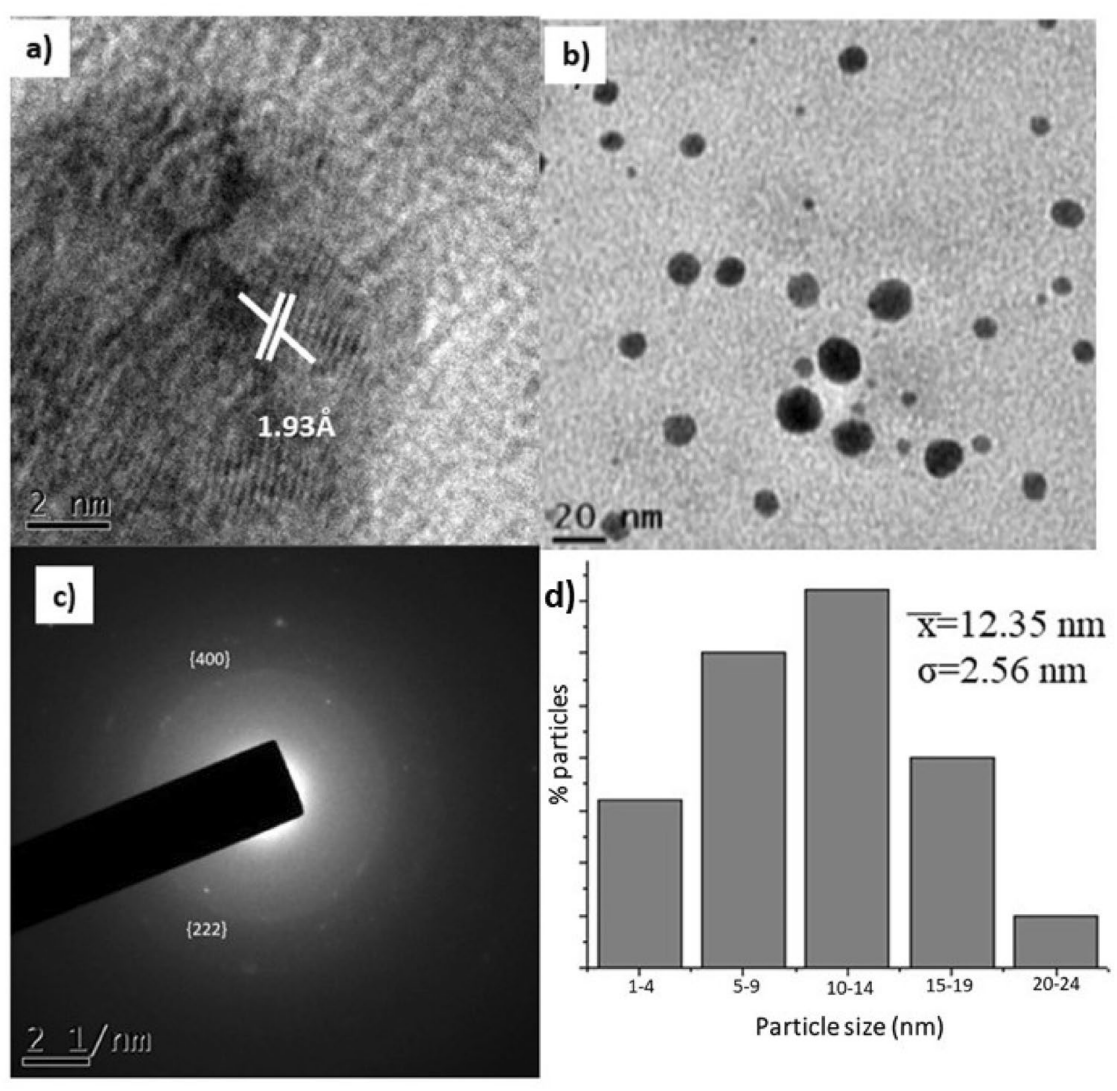
Transmission Electron Microscopy (TEM) micrographs; (**a**) corresponding high-resolution analysis by Transmission Electron Microscopy (HRTEM), (**b**) analysis biogenerated AgNPs (**c**) Selected Area Electron Diffraction (SAED), and (**d**) average nanoparticle diameter histogram.

In the TEM micrographs we can observe that through a high-resolution analysis (HRTEM) in Fig. 3a, the crystalline structure of the AgNPs was corroborated with the measurements of the interplanar distance of 1.93 Å, which corresponds to the [200] plane. Regarding Fig. 3b it is clearly shown that biogenic AgNPs are predominantly nearly spherical. Selected area electron diffraction (SAED) (Fig. 3c), indicated that the measurements of the family of crystalline planes correspond to the metal Ag and to the FCC (Face Centered Cubic) crystalline structure, which we corroborate from JCPDS card 00-004-0783. The size distribution histogram of AgNPs ranged from ∼2–24 nm ($\overline{X }$ = 12.35 nm, σ = 2.56 nm, Fig. 3d).

Regarding the characterization of the size, it was found by DLS (dynamic light scattering) that the hydrodynamic size is 23.45 nm, while the polydispersity index (PDI) was 0.242.

**Figure 4:**
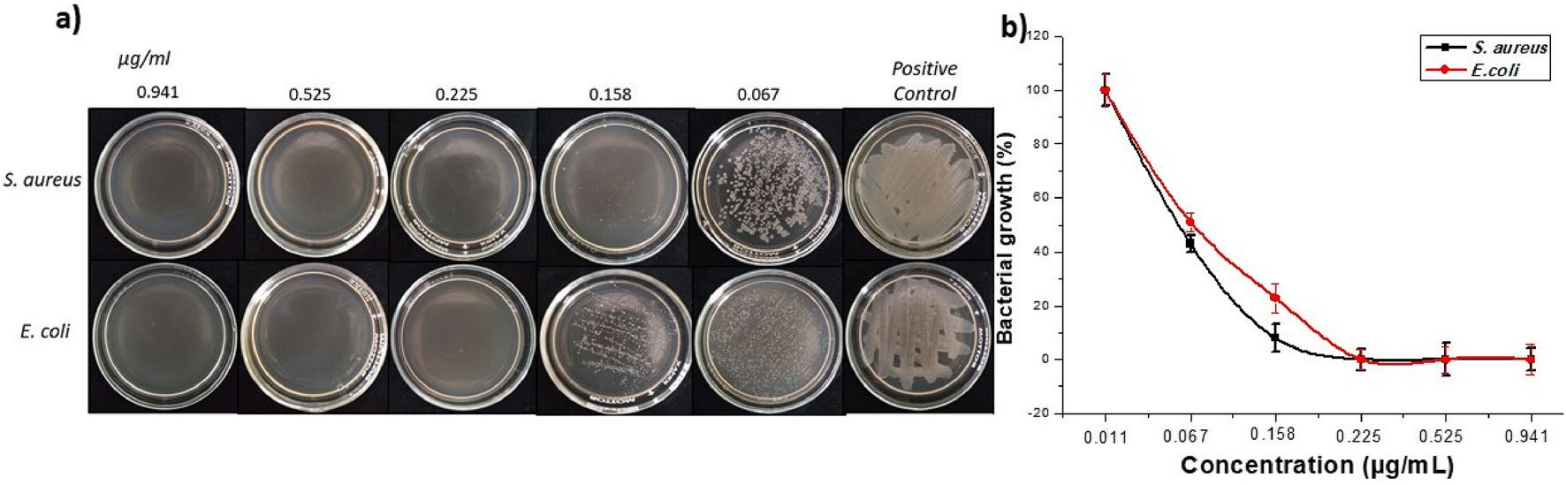
Antimicrobial assay; (**a**) Antimicrobial test of silver nanoparticles against *E. coli* and *S. aureus* (**b**) Graph of microbial growth with respect to different concentrations of nanoparticles.

The results of the "broth microdilution" test are shown in Fig. 4, to evaluate the antibacterial activity of the AgNPs, the pathogenic strains of *E. coli* and *S. aureus* were used. AgNPs were effective against both strains. In *E. coli*. the maximum zone of inhibition was at a concentration of 0.225 µg/ml. while for *S. aureus* it was 0.158 µg/mL, as shown in Table 1.

**Table 1:**
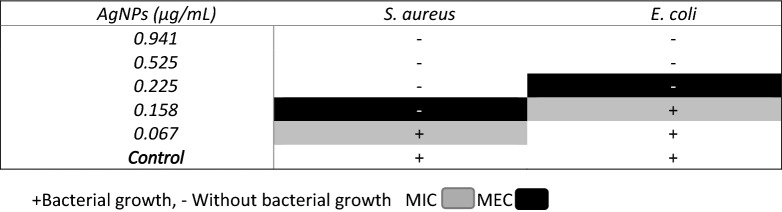
Minimum inhibitory concentration and maximum effective concentration of AgNPs.

**Figure 5:**
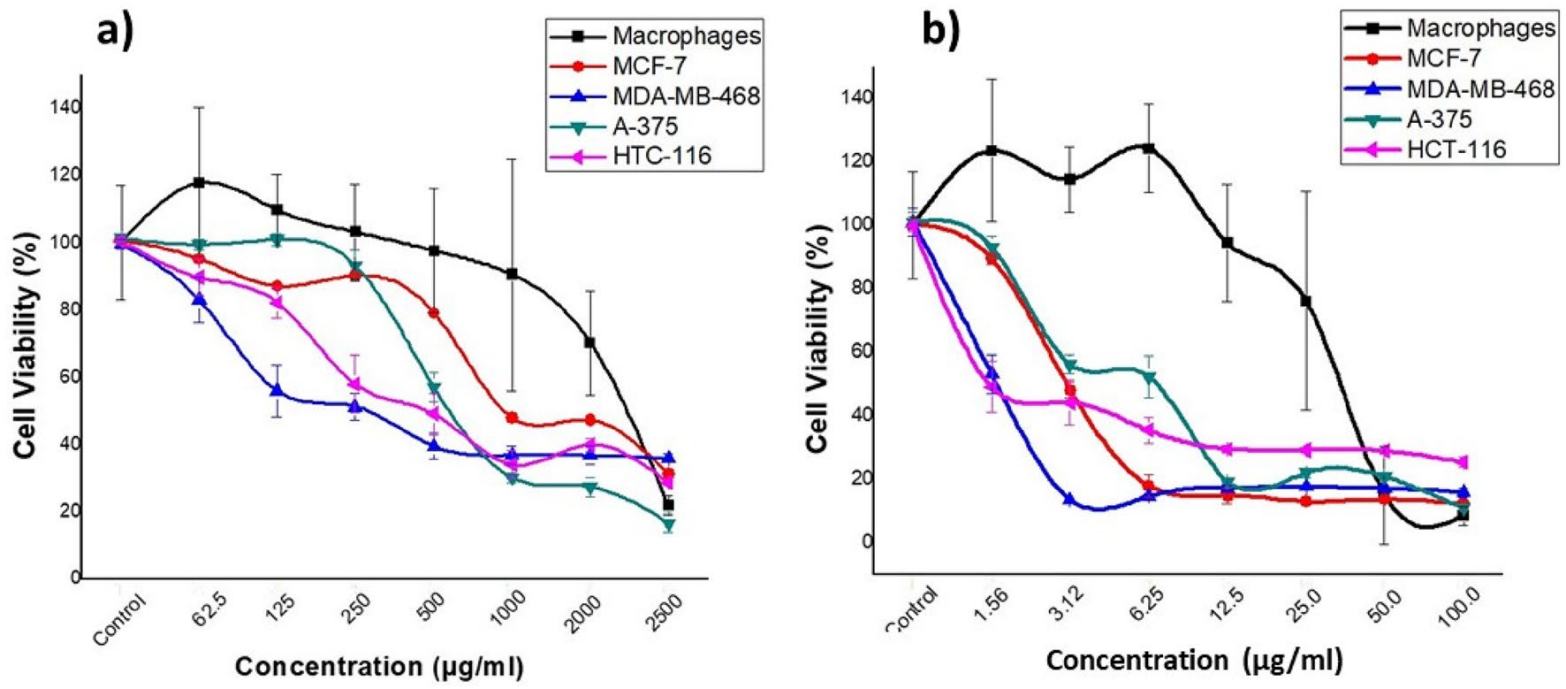
Graphs of the percentage of cell viability with respect to different concentrations of: (**a**) extracts of *C. procumbens* and (**b**) silver nanoparticles (AgNPs).

**Figure 6:**
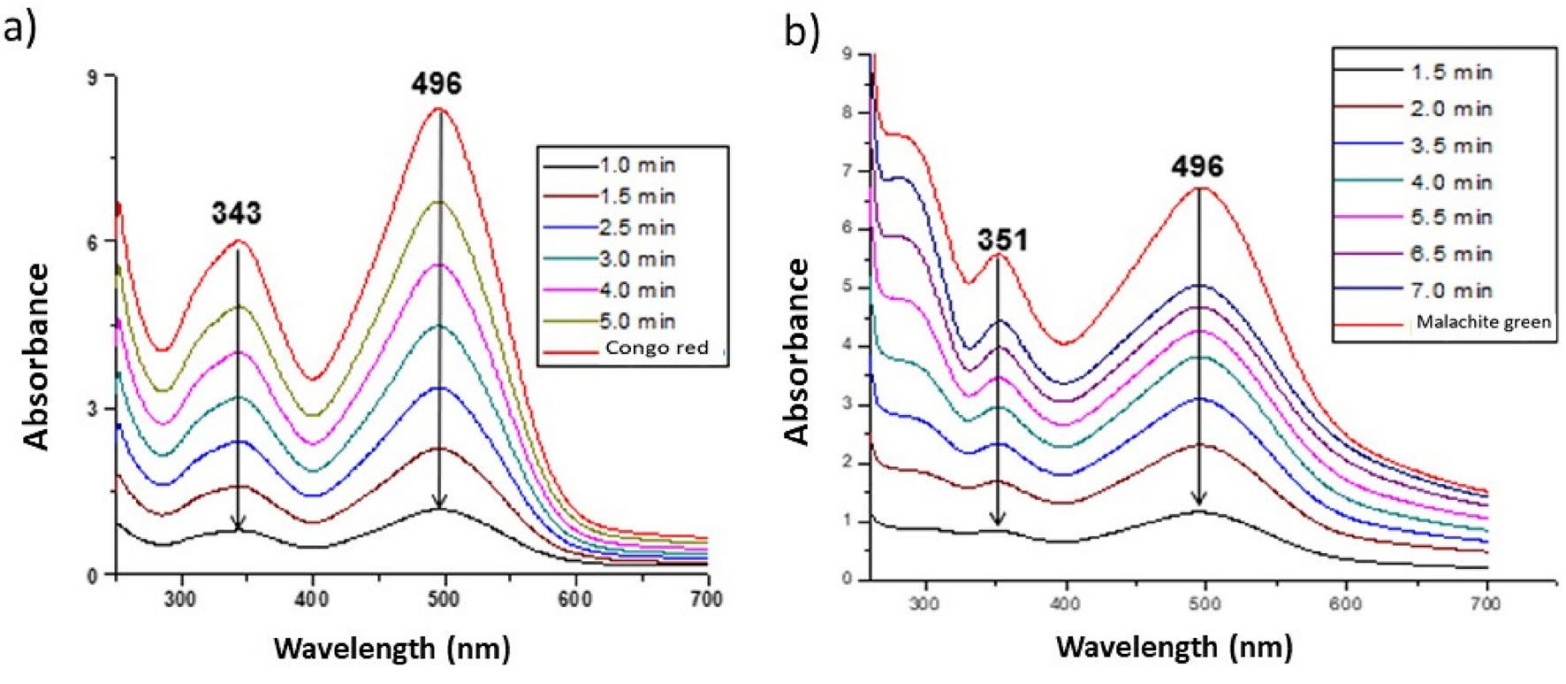
Study of kinetics of the (**a**) congo red and (**b**) malachite green dye degradation by the synthesized AgNPs.

To evaluate the antitumor effect of AgNPs and *C. procumbens* extracts, cell viability assays were performed with colon cancer cell lines (HCT-116), melanoma (A-375), breast cancer (MCF-7 and MDA-MB-468) and macrophages (Fig. 5). As shown in Fig. 6 and Table 2, AgNPs effectively reduced cell viability in all cancer cell lines, AgNPs effectively reduced cell viability in all cancer cell lines, with values of 1389, 6.515, 2.556, 2250 for HCT-116, A-375, MCF-7 and MDA-MB-468 cell lines, respectively.

**Table 2:** Antiproliferative activities (IC_50_ (µg/mL)) of AgNPs and *C. procumbens* extracts in cancer cell lines and macrophages. All the experiments were performed in triplicate.

Treatment	MCF-7		MDA-MB-468		A-375		HCT-116		Macrophages	
	IC_50_	r^2^	IC_50_	r^2^	IC_50_	r^2^	IC_50_	r^2^	IC_50_	r^2^
Agnps	2.566	0.9345	2.250	0.9132	6.515	0.9928	1.389	0.8903	70.950	0.9454
*C. Procumbens* extract	687.5	0.9678	314.5	0.9793	935.4	0.9298	492.5	0.8980	71.344	0.9239

The percentage of viability decreased with increasing concentrations of the plant extract (Fig. 5a). The IC_50_ of the extract are higher than IC_50_ of AgNPs (Table 2). As expected the AgNPs potentiated the effect (Fig. 5b) as it has been observed by other research groups^46^. Likewise, the therapeutic index values indicated that the effect of the AgNPs is higher than the extract alone (Table 3).

**Table 3:** Therapeutic indexes (µg/mL) for AgNPs and *C. procumbens extract* for different tumor cell lines compared to the macrophage cell line.

Treatment	MCF-7	MDA-MB-468	A375	HCT-116
AgNPs	27.65	31.53	10.89	51.07
*C. procumbens* extract	0.103	0.226	0.076	0.144

The ability of the AgNPs to degrade congo red and malachite green dye of the synthesized AgNPs is presented in Fig. 6. Congo red and malachite green are non-biodegradable and toxic azo dyes that can be degraded through the use of NPs. The degradation reaction of congo red and malachite green was monitored by UV–Vis spectrophotometer. Congo red in water showed a band at 496 nm and an electron transition of 343 nm associated with the azo group. While the malachite green degradation reaction showed an SPR band at 496 nm and an electron transition of 351 nm. From these graphs, it is observed that the absorption peak of the dye molecules gradually decreased over time. The absorption peak disappeared and the color of the solution changed from red to colorless.

**Figure 7:**
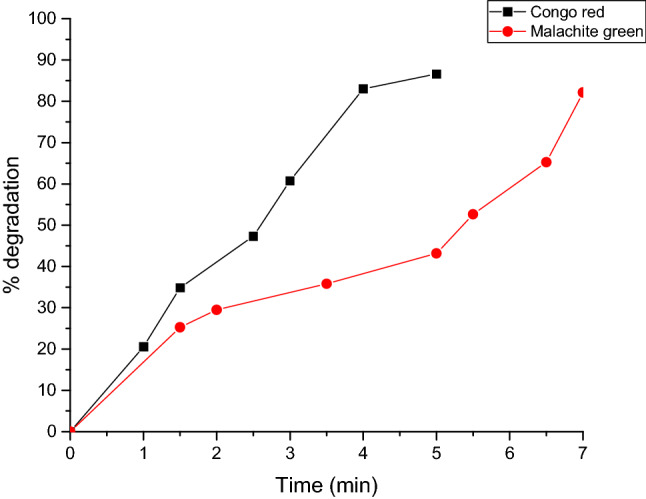
Graph of the percentage of degradation of the dye Congo red and malachite green with respect to time.

Regarding the degradation percentages, we observed a value of 86.61% for congo red and 82.11% for malachite green, observing a significant decrease in dye degradation from the first contact (Fig. 7).

## Discussion

According to the potential the AgNPs can be used in three main application including degradation of dyes, antibacterial tests and antitumor agents. The formation of AgNPs was monitored^47^, observing a change in color of the reaction mixture that began to change from a light yellow to dark brown after 6 h, which indicated the generation of AgNPs, due to the participation of a redox reaction of silver metal Ag^+^ ions in silver Ag° nanoparticles through the active molecules present in the extract of C. procumbens48. This color is attributed to the excitation of SPR49. A characteristic and well-defined SPR band for silver nanoparticles was obtained at around λ 441 nm^7^.

FTIR measurements of biosynthesized AgNPs were performed to identify possible biomolecules responsible for the stabilization of AgNPs. Previous studies have revealed that carbonyl, amide, and amino groups show a tendency to bond with metal particles. This helps to form a layer on the metallic nanoparticles, which ensures their stabilization and agglomeration. The amide and other functional groups in the extract can probably influence the interaction of AgNPs with peptides or carbohydrates, thus stabilizing them. The smaller size and crystal structure of AgNPs have excellent antimicrobial potential^50^. TEM analysis of the particles provides information on size and formation. The mean sizes of polydispersed silver AgNPs have been shown to be 20 nm. TEM images of silver nanoparticles have shown that the morphology of silver nanoparticles was predominantly quasi-spherical with a mean diameter of 12.3 nm and a standard deviation 2.56 nm. These results are similar to those obtained by other authors using the same methodology^51–53^. This is due to the bioreduction from various compounds in the medium, as commonly occurs in green synthesis. In addition, it is possible to corroborate the crystal structure of the AgNPs, in fact it coincided with what is specified by card 00-004-0783^54^ with an analysis corresponding to the micrographs of HRTEM and SAED, with this analysis it is determined that AgNPs are indeed composed of the element Ag (FCC). The data obtained from the DLS measurements of hydrodynamic size and polydispersity index (PDI) were 23.45 nm and 0.242 respectively. As can be seen there is a difference of 11.15 nm ± 2.56, this is due to that only the nanoparticle count was made through a small part of the copper grid while the DLS technique is more precise and takes into account the sample in solution without eliminating the medium in which the nanoparticles are suspended. The minimum inhibitory concentrations (MIC) values of AgNPs against *E. coli* and *S. aureus* were obtained to determine the lowest concentration of AgNPs that can cause the inhibition of the growth of the bacteria *E. coli* and *S. aureus*. Cultures of *E. coli* and *S. aureus* without nanoparticle treatment were used as controls; AgNPs showed the highest activity against *E. coli* with a MIC value of 0.225 mg/mL, while for *S. aureus* the MIC value was 0.158 mg/mL. This indicates that the AgNPs biogenerated in this study were more selective towards Gram-negative bacteria (*E. coli)* than Gram-positive bacteria (*S. aureus)* (Fig. 4).

AgNPs activity is highly dependent on AgNPs concentration. This is in accordance with what was reported in a previous work^55^. The interface between bacteria and AgNPs can generally be described by the following approaches: first, nanoparticles possess an extremely large surface area that provides better contact and interaction with bacterial cells^18^. Second, their interactions may be between positively charged membrane proteins present on the surface of bacteria and negatively charged AgNPs^56^. Furthermore, the attraction of these NPs to the surface of the cell membrane depends on the particle's surface area. The smaller size of the AgNPs will offer a better surface area that can interact and penetrate the membrane of the cell surface. This will provide a significant bactericidal effect and cause bacterial cell death^56^.

Likewise, it is possible to observe a very important principle; apparently our generated nanoparticles, unlike the extract, exert their antiproliferative effect on tumor cells with much lower concentrations that go unnoticed by macrophages. This fact is of great importance as it could prevent many of the side effects of current cancer treatments. It may be due to that AgNPs are going to sites with greater energetic activity, such as tumors or cancer cells^57^.

It is important to recognize that the biogenerated AgNPs have a powerful antitumor effect on cell lines from various tissues, highlighting its effect on the colon cancer line HCT-116 (IC_50_ = 1.389; TI = 51.07). This effect is comparable with those obtained with *C. colosynthis extracts*^58^. Moreover, our results showed that IC_50_ values exceed the effect of other plants as a reducing agent. For example Sijo Francis presents values of 15.68 μg/mL for AgNPs synthesized based on *E. scaber* on the A-375 cancer cell line^46^. Similarly, Jannathul Firdhouse M. & Lalitha P. reported IC_50_ values of 3.04 μg/mL for AgNPs biosynthesized with *Alternanthera sessilis* against the MCF-7 cancer cell line that are higher doses that the ones we obtained^59^. Other studies also showed the IC_50_ of *d Fumaria parviflora* extract and AgNPs- *d Fumaria parviflora* against MDA-MB-468 cancer cells were observed at 100 µg/mL and 80 µg/mL respectively^60^. Therefore, it is assumed that the potentiated effect of the nanoparticles is due to the component of the *C. procumbens* extracts (Table 2) that is working as a reducing and stabilizing agent of the AgNPs. We observed that the concentration of the extract used for the biogeneration of nanoparticles is sufficient to generate antitumor activity, bioreduce nanoparticles and stabilize them. It should be noted that these results depend greatly on the concentration of metabolites present in the extracts and on the concentrations of the precursor salt used^61^. If we compare with recent studies of AgNPs synthesized by chemical methods, such as the nanoparticles synthesized by Al-Khedhairy, AA and Wahab, R.^62^ in which they report an IC_50_ value of 9.85 µg/mL, as we can see the IC_50_ values are more lower than even the values provided by a chemical synthesis. That makes our research generate greater interest. We attribute this important property to the potentiation of the effect of the AgNPs and the aqueous extract of *C. procumbens* as a whole.

The importance of our work relies in the therapeutic index (TI) obtained, since its value was high for all tumor cell lines treated with AgNPs compared to the extract (Table 3). The best TI was achieved with HCT-116 and MDA-MB-468 (TI = 51.07 and 31.53 respectively). In the specific case of the MCF-7 cell line, a value of 27.65 was obtained. and it was compared with *Melia dubia*^63^ and *Cassia fistula*^64^, which present a therapeutic index of 16.02 and 9.23 respectively,. Additionally, human lung epithelial carcinoma cells (A549) and human breast epithelial cells (HBL100) have been studied through the therapeutic index with silver nanoparticles obtained with plant extracts, obtaining a therapeutic index of 2.^63^. In human cervical cancer (HeLa) cells, metal nanoparticles generated from green synthesis have had therapeutic indices of ≤ 2.5^65^. In addition, we have seen that a similar concentration of the extract and the AgNPs induces inhibition of 50% of macrophage proliferation. Hence, we suspect that tumor cells are less susceptible to damage from flavonoids present in the aqueous extract of *C. procumbens*, while AgNPs enhance its antitumor effect. This is due to the rapid proliferation of cancer cells, since they require high levels of energy, so they can be more sensitive and have important physiological changes^66^.

Congo red is a diazo-anionic dye^67^. This colorant, in addition to affecting the aesthetics, the transparency of the water and the solubility of oxygen in the bodies of water, has been reported as highly toxic for living beings because it causes carcinogenesis, mutagenesis, teratogenesis, respiratory damage, allergies and problems during pregnancy^68^. The congo red or salt of 3,3'- (4,4'-biphenylenebis (azo) bis (4-amino) disodium naphthylene sulfonic acid is prepared by a tetradiazotization with benzidine and naphthylsulfonic acid. The covalent bonds in the molecule confer stability, which together with the complex molecular structure they make biodegradation and photodegradation difficult. Congo red in an aqueous solution (distilled water) shows a Surface Plasmon Resonance (SPR) band at 496 nm (π → π) and the electronic transition at 351 nm (north → π) is associated with the azo group. In Fig. 6a it is observed that the absorption peak of the dye molecules gradually decreases in a time dependent manner. The solution fades from red to colorless. Malachite green is a cationic triphenylmethane dye that is widely used in various fields as a parasiticide. The catalytic degradation of the malachite green organic dye was controlled by the change in the absorbance of ultraviolet light. In addition, the degradation capacity in liquid medium was evaluated (Fig. 6b)^69^. According to the literature, the photocatalytic activity^33^ of NPs depends on the shape, size and crystalline structure of particles^69^. To our knowledge, this is the first time that biosintethized AgNPs are able to degrade this colorant at a low concentration. Our results are really very promising, compared to other authors such as Lateef and Akande^70^, who only reach 80% degradation with 20 µg/mL^70^, which is interesting because the concentration of nanoparticles used in our study is much lower. Even other authors report that using NaBH_4_ manage to degrade colorants in a longer period of time^71^, comparing our results it is important to highlight that we are not using any type of additional catalyst^72^, nor radiation or exposure to light, in addition to applying other techniques^73^.

## Methods

### Biosynthesis

#### Preparation of the extract of *C. procumbens*

For the elaboration of the aqueous extract, leaves of *C. procumbens* were collected in the month of May in the municipality of Villa Victoria, State of Mexico, Mexico, handling of plants were carried out in accordance with Mexican guidelines and regulations, washed thoroughly with distilled water, allowed to dry at room temperature for 15 days, chopped finely and ground, in 100 mL of sterile distilled water, 0.5 g of the treated leaves of *C. procumbens* were added and heated to boiling point for 5 min. Then was filtered the extract through Whatman No. 1 filter paper (size of 25 µm pore). A second filtration step was carried out using Amicon Ultra-15 30 kDa tubes. To purify the aqueous extract, we performed a second filtration step using an Amicon 30 kDa ultrafiltration unit. The ultrafiltration units were centrifuged at 300 rpm for 10 min.

#### Synthesis of silver nanoparticles

For the synthesis of nanoparticles, a 0.001 M aqueous solution of Silver Nitrate AgNO_3_ (Sigma-Aldrich) and an aqueous solution of *C. procumbens* extract were used. With a volume-to-volume ratio of 1:1, that is, 5 mL of aqueous extract of *C. procumbens* as reducing agent and 5 mL of silver nitrate precursor salt as oxidizing agent. The silver ions were reduced to metallic silver within 6 h, showing a color change from light to dark brown.

### Characterization of AgNPs

#### UV–Vis spectroscopy

UV–Vis spectroscopic analysis was performed to monitor the formation of AgNPs using a UV–Vis spectrophotometer (VE-5100UV spectrophotometer, USA)^28^. UV–Vis adsorption spectra were measured in a 1 cm quartz cuvette using 2 mL of the synthesized AgNPs solution. The samples were measured in wavelength ranges between 350 and 750 nm.

#### Catalytic degradation of congo red and malachite green dye

An aqueous solution of the malachite green and congo red dyes (10^–3^ M) was prepared. Then 0.1 ml of AgNPs (0.941 µg/mL) solution was added to 2 mL of the dye solution. The dye degradation experiments were carried out under shaking and irradiation of a solar simulator (ScienceTech SF150B). The degradation of the solution was followed by measuring the absorption band characteristic of dye, using a UV–Vis spectrophotometer (VE-5100UV spectrophotometer, USA). To obtain the percentage of degradation: the dyes and the AgNPs were kept in linear agitation during the degradation period, additionally Eq. (1) was used.

1 $$\% degradation=\frac{{A}_{0}-{A}_{t}}{{A}_{0}}x100$$

where A_0_ is the initial absorbance of the dye and A_t_ is the absorbance of the dye at a specific time.

#### FTIR analysis

FTIR spectrums of the biogenic AgNPs and plant extracts were obtained in a Perkin Elmer spectrophotometer (L16000300 Spectrum Two LiTa, Llantrisant, UK), using the potassium bromide (KBr) pellet method. The samples were measured in wavelength ranging between 500 and 4000 cm^−1^.

#### TEM analysis

Morphology and size distribution of AgNPs were investigated using a JEOL-2100 High-Resolution Transmission Electron Microscope (HR-TEM). Samples were prepared by placing a drop of AgNPs, dispersed in solution, followed by solvent evaporation.

#### Characterization of the size and zeta potential

The hydrodynamic mean diameter of the AgNPs was determined by photon correlation spectroscopy (PCS), using a 4700 C light-scattering device (Malvern Instruments, London, UK) working with a He–Ne laser (10 mW). The diffusion coefficient measured by the dynamic light scattering was used to calculate the size of the AgNPs by means of the Stokes–Einstein equation. The homogeneity of the size distribution is expressed as polydispersity index (PDI), which was calculated from the analysis of the intensity autocorrelation function (Zeta-Sizer Nano Z, Malvern Instruments, UK).

### Broth dilution test

Experiments on antimicrobial or antifungal activity were performed as described by the Institute for Clinical and Laboratory Standards (Wikler, 2009). AgNPs were tested against human pathogenic strains such as *S. aureus ATCC25293* and *E. coli* ATCC25922 by determining the minimum inhibitory concentration (MIC) and the minimum effective concentration (MEC) following the broth microdilution method (Kim and Anthony, 1981). Bacteria were cultured to prepare 0.5 McFarland standards. Mueller–Hinton broth medium (100 µl) was placed in each well and 100 µl AgNPs was added and then serial dilutions were made starting from the first row. A negative control (broth and microorganisms) and a sterile control (broth only) were used. Each well was then aseptically inoculated with 5 µl of the microorganism suspension (the final concentration was approximately 5 × 10^5^ CFU/mL). Assays were performed in triplicate for each concentration and strain. The inoculated microplates were incubated at 37° C with continuous shaking at 200 rpm for 24 h. Subsequently, the colonies were counted and the MIC and MEC were determined.

### Cell viability assay

Breast cancer (MCF-7, MDA-MB-468), colon cancer (HCT-116), Melanoma (A-375) cell lines and macrophages were obtained from the Biobank (Sistema Sanitario Publico Andaluz, Granada, Spain). Cells were grown in Dulbecco’s Modified Eagle’s Medium (DMEM) (Sigma, St. Louis, MO, USA) supplemented with 10% fetal bovine serum (FBS) and 1% penicillin/streptomycin (P/S) (Sigma, St. Louis, MO, USA). The cells (5 × 10^3^ cells) were seeded in 96-well plates at 37 °C in a 5% CO_2_ atmosphere and 24 h later, the cells were treated with different concentrations of biosynthesized AgNPs and incubated for 3 days. Then, the medium was removed and the cells were washed with PBS. Subsequently, 100 μL of 3-(4,5-dimethyl-2-thiazolyl)-2, 5-diphenyl-2-tetrazoyl bromide (MTT) (at a concentration of 0.2 mg/mL) was added to each well and incubated for three hours. After this time, the MTT reagent was removed, and the formazan crystals formed were dissolved by adding 100 μL of dimethyl sulfoxide (DMSO) per well and analyzed at 570 nm in a multi-well ELISA plate reader. The inhibitory concentration 50 (IC_50_) was calculated with the GraphPad Prism program (GraphPad 6 Software San Diego, CA, EE. UU). All the experiments were plated in triplicate and were carried out at least twice. In addition, non-treated cells were used as controls. The therapeutic index was calculated for both the extracts and the AgNPs, from Eq. (2). The therapeutic index is expressed numerically as a ratio between the dose of the drug that causes death (lethal dose or DL) or a deleterious effect in a proportion "x" of the sample and the dose that causes the desired therapeutic effect (effective dose or DE) in the same or greater proportion "y" of the sample.

2 $$TI=\frac{\mathrm{DL}50}{DE50}$$

#### Statistic analysis

The data collection from the different biological studies represents the mean ± standard deviation. The two-tailed Student's t-test was used to compare the differences between two groups. A two-tailed p value < 0.05 is considered statistically significant.

## Conclusions

Biogenerating silver nanoparticles from natural products such as the aqueous extract of *C. procumbens* is an environmentally friendly method, does not produce unwanted contaminants and is very easy to reproduce. Our results show that biogenerated AgNPs has potential as a microbial agent, anticancer agent, and also opens the possibility for the degradation of specific dyes. It is a simple procedure with several advantages such as cost-effectiveness, biocompatibility for medical applications, as well as large-scale commercial production. These results give us an opening to continue investigating the applications that we will surely be reporting in future works.

## Data Availability

All data generated or analysed during this study are included in this published article.
